# Impact of Built-In Software Monitoring on Survival in Amyotrophic Lateral Sclerosis Patients Receiving Home Mechanical Ventilation: A Cohort Study

**DOI:** 10.3390/jcm15041513

**Published:** 2026-02-14

**Authors:** Ana Hernández-Voth, Javier Sayas-Catalán, Marta Corral-Blanco, Miguel Jiménez-Gómez, Gema Carvajal-Cuesta, Manel Luján-Torné, Cristina Lalmolda-Puyol, Pablo Florez-Solarana, Victoria Villena-Garrido

**Affiliations:** 1 Mechanical Ventilation Unit, Pulmonology Department, 12 de Octubre University Hospital, 28041 Madrid, Spain; anahvoth@gmail.com (A.H.-V.); jsayascat@gmail.com (J.S.-C.); m.corralblanco@gmail.com (M.C.-B.); gcarva.cuesta@gmail.com (G.C.-C.); vvillena@separ.es (V.V.-G.); 2 Respiratory Diseases Research Group, 12 de Octubre Hospital Research Institute (I+12), 28041 Madrid, Spain; 3 Department of Medicine, Universidad Complutense de Madrid, 28040 Madrid, Spain; 4 Pulmonology Department, Corporació Sanitari Parc Taulí, 08208 Sabadell, Spain; mlujan@tauli.cat (M.L.-T.); clalmolda@tauli.cat (C.L.-P.); florezsolarana@gmail.com (P.F.-S.); 5 CIBER de Enfermedades Respiratorias (CIBERES), 28029 Madrid, Spain

**Keywords:** amyotrophic lateral sclerosis, mechanical ventilation, built-in-software, prognosis

## Abstract

**Background/Objectives**: Amyotrophic lateral sclerosis is a progressive neurodegenerative disease in which respiratory failure is the leading cause of death. Mechanical ventilation improves both survival and quality of life; however, the prognostic implications of built-in ventilator software monitoring remain insufficiently characterized. The aim of the study was to determine whether built-in ventilator software-based monitoring is associated with enhanced survival in amyotrophic lateral sclerosis subjects. **Methods**: Cohort study of amyotrophic lateral sclerosis subjects, stratified into two groups: those monitored through detailed built-in ventilator software and those not monitored. Clinical and ventilatory data were systematically evaluated during a 24-month follow-up. **Results**: Among 120 ALS subjects (57 detailed built-in ventilator software, 63 non-detailed ventilator software), median survival from diagnosis was significantly longer in the detailed built-in ventilator software group (3.42 vs. 2.12 years; *p* < 0.001). Survival from mechanical ventilation initiation was also significantly longer in the built-in ventilator software group (2.79 years vs. 0.78 years). Greater daily mechanical ventilation usage was associated with shorter survival (*p* < 0.003). Paradoxically, subjects with the lowest proportion of spontaneous inspirations exhibited superior survival outcomes (*p* = 0.04). Neither persistent leaks nor asynchronies were independently predictive of survival. **Conclusions**: BVS-monitoring was associated with improved survival in amyotrophic lateral sclerosis subjects receiving home mechanical ventilation. Its integration into clinical practice may enable timely, data-driven ventilatory adjustments, ultimately contributing to more individualized and optimized patient management.

## 1. Introduction

Amyotrophic lateral sclerosis (ALS) is a progressive degenerative disorder typically leading to death from respiratory failure within 2–4 years of symptom onset. Multiple prognostic determinants have been described, including advanced age [1], bulbar onset [2,3,4,5], diagnostic delay [2], rate of disease progression [6], creatinine [7], development of frontotemporal dementia [8], nutritional status [2], and functional impairment measured by the ALSFRS-R scale [8,9,10,11]. Additional factors include follow-up within a multidisciplinary care model unit [12] and the extent of respiratory involvement [1,2,3], the latter being profoundly modified by the introduction of mechanical ventilation (MV).

MV improves survival [4,13,14] but also enhances quality of life [4,15,16,17]. Therefore, respiratory failure represents a pivotal determinant of prognosis, primarily managed with non-invasive MV and, in selected cases, invasive MV. Recent advances in MV technology have incorporated built-in ventilator software (BVS) capable of continuously recording ventilatory parameters [18,19,20], including patient–ventilator synchrony, adherence patterns, unintentional leaks and respiratory events, offering a valuable opportunity to refine ventilatory support and guide clinical decision-making [21].

Although BVS has been increasingly adopted in routine practice, its prognostic significance in ALS has not been formally evaluated. This study aims to determine whether BVS monitoring is associated with improved survival and to identify specific BVS-derived variables with potential prognostic value in ALS subjects receiving home MV.

## 2. Materials and Methods

### 2.1. Study Design and Patient Selection

The study included subjects diagnosed with ALS followed at the Neuromuscular Multidisciplinary Unit of a tertiary national referral hospital. The study adhered to STROBE guidelines [22]. Bulbar versus spinal onset was determined clinically according to the ALSFRS-R, based on the bulbar sub-scores (speech, salivation, swallowing).

Inclusion criteria were (1) a diagnosis of ALS according to the revised El Escorial criteria [23]; (2) eligibility criteria for the initiation and follow-up of home MV, based on national guidelines (Figure S1); and (3) regular follow-up visits every 3 months during MV support. Exclusion criteria included (1) diagnosis of atypical ALS variants (e.g., primary lateral sclerosis, progressive muscular atrophy, or ALS plus syndromes); (2) MV initiated during emergency visits for acute, non-neuromuscular conditions such as infections or cardiopulmonary decompensation [5]; (3) non-adherence to scheduled follow-up visits; and (4) insufficient ventilator use (less than 4 h per day on average). To reduce selection bias, all subjects who fulfilled these criteria during the study period were included.

Subjects were classified into two groups according to the monitoring strategy. The non-BVS group was monitored using standard clinical assessment and basic ventilator information (mean usage, tidal volume and leaks), whereas the BVS group underwent systematic BVS monitoring, including regular data downloads and interpretation of summary parameters (mean usage, respiratory rate, percentage of spontaneous inspiratory cycles, tidal volume and residual apnea–hypopnea index) and flow-pressure waveforms at each visit. Group allocation was period-based: the BVS group included subjects prospectively followed after the implementation of BVS as standard practice (February 2017–December 2020), while the non-BVS group comprised retrospectively identified subjects followed before BVS implementation.

Patient data were collected at eight consecutive visits over approximately 24 months after the start of MV, or earlier in case of death or tracheostomy. To minimize loss to follow-up, when patients were unable to attend scheduled clinic visits due to disease progression, caregivers were encouraged to attend the visit and bring the ventilator device to download BVS data. As a result, decreases in sample size across visits were exclusively due to death or tracheostomy, and no participants were lost to follow-up for non-medical reasons (Figure S2). Pulmonary function tests were not routinely performed after MV initiation because, in ALS subjects—particularly those with bulbar dysfunction, they are often not feasible or reliable and provide limited additional information once MV is established. In subjects with symptoms suggestive of hypoventilation, transcutaneous capnography was performed; however, systematic nocturnal/diurnal oximetry or continuous transcutaneous carbon dioxide monitoring were not routinely applied.

### 2.2. Ventilator Adjustments and Clinical Management

All subjects were ventilated using standard pressure-support spontaneous/timed (S/T) modes, with availability of BVS analysis. No automated methods were used. Titration was performed on an outpatient basis with the patient in the supine position, and initial settings were individualized based on tolerance, comfort, and real-time analysis of flow–pressure curves. Settings generally started with low pressures (PEEP 6–8 cmH_2_O, IPAP 10–14 cmH_2_O), adjusted based on waveform analysis and clinical signs of under-assistance, rather than targeting a predefined tidal volume or respiratory rate. Trigger sensitivity was generally set at moderate-to-high sensitivity. Ventilator settings were subsequently optimized based on improvement in orthopnea and hypoventilation related-symptoms and on correction of leaks and patient–ventilator asynchronies, ensuring adequate patient tolerance.

Although no predefined numerical targets were used, persistent abnormalities in monitored parameters systematically prompted specific interventions. Persistent unintentional leaks (typically >20 L/min) led to interface optimization and pressure support reduction in well-ventilated patients, if tolerated. In case of residual events (>10 events/h) or obstructive events identified on the detailed flow-pressure waveforms, PEEP was manually increased by the clinician and reassessed at subsequent visits. If patient–ventilator asynchronies were identified (e.g., ineffective efforts or apnea–hypopnea events), targeted individualized interventions following the same principles were conducted across all devices. Adjustments included changes in trigger sensitivity (in case of ineffective triggering), pressure support (in case of under-assistance asynchronies), PEEP (in case of air trapping) and cycling percentage (in case of premature or delayed cycle), corrected based on the asynchrony. In daily practice, inspiratory time range (minimum and maximum) for spontaneous breaths was only modified in selected cases with significant unintentional leaks or patient’s discomfort. The backup respiratory rate was not modified during follow-up unless there was ventilatory insufficiency or intolerance. Instead, the progressive decline in spontaneous inspiratory efforts, resulting in a greater proportion of breaths delivered at the fixed backup rate, was used as an indirect marker of disease progression. All modifications were made according to best clinical practices, based on clinical evaluation and BVS-derived data.

In contrast, subjects in the non-BVS group were managed based on clinical judgment, patient-reported symptoms and basic ventilator information available directly on the device display (e.g., average tidal volume, total leaks, and daily usage) [24]. The presence of abnormal values of tidal volume raised the suspicion of leaks, leading to the assessment of the interface. After the exclusion of significant leaks, pressure support was adjusted.

The ventilatory mode remained static throughout follow-up, and adjustments were limited to manual changes in pressure support, PEEP, trigger sensitivity, or interface fitting when clinically indicated.

### 2.3. Statistical Analysis

Normality was assessed using the Kolmogorov–Smirnov test. Between-group comparisons for continuous variables were conducted using Student’s *t* test or the Mann–Whitney U test, depending on distribution. Categorical variables were analyzed using chi-square or Fisher’s exact tests.

Survival was analyzed using Kaplan–Meier curves and compared with the log-rank test. Survival time was calculated from the date of ALS diagnosis. A secondary survival analysis was performed using the initiation of home MV as time zero to enhance comparability between groups. Subjects who underwent a tracheostomy (*n* = 3) were censored at the date of the procedure, as the study endpoint was survival under non-invasive ventilation. Missing BVS data at individual visits were handled using an available-case approach without imputation. As noted in the Methods section, attrition across visits was driven exclusively by death or tracheostomy.

A two-tailed *p* value < 0.05 was considered statistically significant. All analyses were conducted using IBM SPSS^®^ version 29.0.

### 2.4. Ethical Approval

The study protocol was approved by the Institutional Research Ethics Committee (protocol CEIm: 18/519). Written informed consent was obtained from subjects prospectively enrolled (BVS group). For retrospective cases (non-BVS group), the requirement for consent was waived in accordance with local regulations. All patient data were anonymized and handled to ensure confidentiality.

## 3. Results

A total of 120 ALS subjects receiving MV were included: 57 in the BVS group (47.5%) and 63 in the non-BVS group (52.5%). During follow-up, 96 subjects (78%) died, all from progressive respiratory failure and disease progression. Tracheostomy was performed in three subjects (2%), all within the BVS group, due to disease progression.

Baseline characteristics were comparable between groups as described in Table 1.

### 3.1. Functional and Ventilatory Progression

The evolution of ALSFRS-R scores, MV usage, spontaneous inspiratory efforts, unintentional leaks, and asynchronies over the eight scheduled visits is summarized in Table 2. Functional status and spontaneous inspirations declined steadily, while ventilator dependence progressively increased, with mean daily usage from 7.5 to 16.2 h/day over time.

The prevalence of unintentional leaks (>20 L/min) remained relatively stable, affecting approximately one-third of subjects at each time point. Asynchronies were observed in 26–53% of subjects, with residual respiratory events being the most frequent type.

### 3.2. Overall Survival and Prognostic Factors

**Figure 1:**
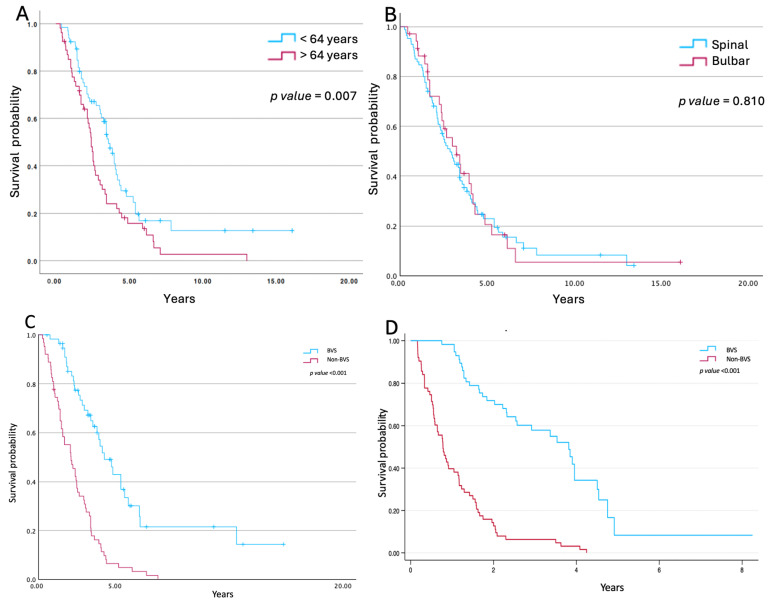
Survival of the studied population according to age at diagnosis (**A**), disease onset (spinal vs. bulbar form) (**B**), survival from diagnosis (**C**), and survival from initiation of MV (**D**) based on BVS or non-BVS follow-up.

Mean overall survival from diagnosis for the entire cohort was 2.60 years (range 0.26–16.10). Age at diagnosis was significantly associated with survival: subjects diagnosed before the median age of 64 years showed longer survival than those diagnosed at an older age (log-rank *p* = 0.008; Figure 1A). In contrast, survival did not differ between subjects with bulbar-onset and spinal-onset disease (median survival 2.58 vs. 2.62 years, respectively; *p* = 0.81; Figure 1B). The time from ALS diagnosis to initiation of mechanical ventilation was similar between groups (median 1.17 years in the non-BVS group vs. 0.92 years in the BVS group; *p* = 0.801).

### 3.3. Association Between BVS Monitoring and Survival

Survival was significantly longer in subjects monitored with BVS. The median survival from ALS diagnosis was longer in the BVS group (3.42 years, 0.55–16.10) than in the non-BVS group (2.12 years, range 0.26–7.84) (log-rank *p* < 0.001; Figure 1C).

To improve comparability between groups, a secondary analysis was performed using initiation of home MV as time zero. Survival from ventilator initiation remained markedly longer in the BVS group, with a median survival of 2.79 years (IQR 1.74–3.85), compared with 0.78 years (IQR 0.46–1.56) in the non-BVS group (log-rank *p* < 0.001; Figure 1D).

A multivariate Cox proportional hazards model was fitted using time from MV initiation as the time scale. BVS monitoring remained independently associated with improved survival after adjustment for age, sex, site of onset and indication of mechanical insuflation-exuflation (hazard ratio 0.18; 95% CI 0.11–0.29; *p* < 0.001) (Table 3).

### 3.4. Prognostic Value of BVS-Derived Ventilatory Parameters

**Figure 2:**
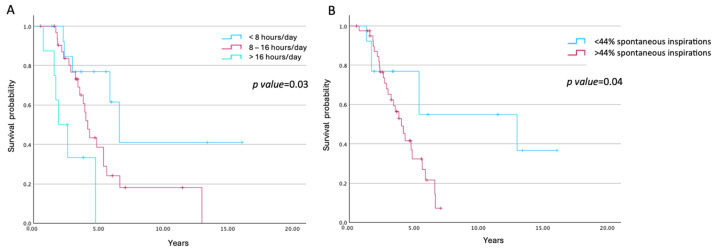
Survival of BVS group according to daily hours of ventilator use (**A**) and percentage of spontaneous inspirations (**B**).

Within the BVS group, ventilator usage at the last follow-up was associated with survival. Subjects were stratified according to average daily use (<8 h/day, 8–16 h/day, and >16 h/day), and survival decreased significantly with increasing ventilator dependence (log-rank *p* < 0.003; Figure 2A).

The proportion of spontaneous inspiratory cycles declined progressively over time, from a mean of 70.2% at the first visit to 38.4% at the eighth visit. When stratified into quartiles, subjects in the lowest quartile of spontaneous inspirations (<44% of cycles) exhibited longer survival compared with those in higher quartiles (*p* = 0.04), a pattern that remained consistent across the full stratification (Figure 2B).

Approximately one-third of BVS group-subjects (Table 2) exhibited unintentional leaks (>20 L/min) at each visit, and up to half experienced asynchronies. Neither persistent unintentional leaks (>20 L/min) nor the presence of patient–ventilator asynchronies were independently associated with survival outcomes (*p* > 0.05).

## 4. Discussion

In this study, we evaluated the impact of BVS monitoring on the survival of a cohort of ALS subjects receiving MV over a 2-year follow-up period. Our findings support the integration of BVS into standard ALS care as a valuable tool for optimizing home MV, particularly through early detection of ventilation-related issues and more individualized adjustments.

Our cohort showed demographic features consistent with previous ALS series [25]. The mean survival of 28 months fits within the wide range reported (13.5–48 months) depending on the study design and patient selection [4,9,14,26,27]. Most studies do not stratify by MV use, although its survival benefit is well-established [28]. The very low tracheostomy rate observed mirrors current European practice, where invasive MV is rarely adopted largely due to patient preferences, cultural context, and healthcare system factors [29,30,31].

Consistent with prior reports, we observed that younger age at diagnosis was associated with improved survival [1,2,3]. Age remains one of the most consistent prognostic markers in ALS, and its impact persists even in cohorts receiving MV [1,2,32] (Figure 1).

By contrast, although bulbar onset is traditionally associated with worse prognosis [2,3,4,5,9,15,33,34], we found no survival differences between bulbar- and spinal-onset subjects. Onset site was defined clinically using the ALSFRS-R bulbar sub-scores, a standard clinical approach in ALS studies, although alternative physiological methods have also been described [35,36,37]. Notably, many of prior reports of worse bulbar outcomes did not account for respiratory failure or MV use. Consistent with previous data [1,14] our data suggest that when respiratory failure is appropriately managed, the site of disease onset does not significantly influence survival. Nevertheless, the lack of association between bulbar symptoms and survival should be interpreted with caution, given the potential selection bias related to device usage.

Although BVS is widely used in clinical settings due to its accessibility, ease of use, and the valuable information it provides [18,20,38,39], its impact on long-term outcomes in ALS had not been formally assessed. BVS-guided management was associated with a median survival extension of approximately 15 months, likely due to more timely ventilator adjustments and individualized care.

When analyzing specific BVS-derived parameters, ventilator usage emerged as a key indicator. In contrast to studies involving heterogeneous neuromuscular populations where increased MV use was linked to better outcomes [40,41], our cohort included only compliant subjects (greater than 4 h/day). Within this group, higher ventilator dependence (particularly usage > 16 h/day) was associated with poorer survival, likely reflecting more advanced disease. The inverse association observed between ventilator use and survival likely reflects underlying disease progression and vital capacity deterioration, rather than a negative impact of ventilator dependence itself.

The proportion of spontaneous inspiration also provided important prognostic insight. As expected, this parameter declined over time, in line with loss of respiratory muscle strength. Paradoxically, subjects in the lowest quartile of spontaneous inspirations (dependent on the ventilator’s backup rate) showed longer survival. In the absence of significant unintentional leaks [38], this may reflect more stable ventilation and reduced respiratory workload, suggesting that full ventilator control in advanced ALS may offer physiological advantages by minimizing asynchronies and fatigue. However, this association should be interpreted with caution, as it may be influenced both by device-specific algorithm artifacts in the classification of spontaneous versus machine-triggered breaths, and by the more intensive optimization of ventilator settings that these highly dependent patients often require.

Unintentional leaks are a well-recognized contributor of asynchrony in MV. However, their detection and quantification can vary depending on the ventilator software used [19,42]. To reduce this bias, leaks were interpreted through longitudinal trends within each patient and by direct waveform inspection, rather than by absolute cross-device values. BVS analysis is considered essential for acute leak estimation [43], with most studies adopting a threshold of >20 L/min to define unintentional or massive leak. In our cohort, approximately one-third of subjects presented with unintentional leaks at each visit, consistent with findings from previous reports. Despite their prevalence, we found no significant difference in survival between subjects with persistent leaks and those without.

Although asynchronies have traditionally been described using polygraphy or polysomnography during MV [21,44], BVS-generated waveforms offer comparable visual information, with the advantage of being accessible at every clinic visit [18,21]. In our cohort, asynchronies were observed in 26% to 53% of subjects (comparable to those reported by Aarrestad et al. [45] (21%) and Carlucci et al. [46] (58%)). The most frequently identified asynchrony was residual respiratory events, present in 16% to 33% of subjects depending on the visit. Notably, this was the only asynchrony consistently observed throughout the entire follow-up period (Figure S3). Residual respiratory events are also the most commonly reported form of asynchrony in the literature [17] and have been associated with worse survival outcomes in ALS [27]. However, other studies have failed to demonstrate a clear link between residual events and prognosis [45], as occurred in our series. This may be explained by the heterogeneous nature of these events—not all represent true obstructions and may be central, due to bulbar dysfunction, or even due to upper airway obstructions due to the retraction of the lower jaw as an effect of the interface [38,47]. Importantly, residual respiratory events are detected through manufacturer-specific proprietary algorithms. To mitigate this variability, our assessment relied not only on longitudinal trends within the same patient, but also on the direct review of airflow and pressure curves. Although subjects requiring PEEP titration had numerically shorter survival, this difference did not reach statistical significance.

This study has several limitations that should be acknowledged. Although it was originally conceived as a prospective randomized trial, the design had to be modified due to the COVID-19 pandemic and the resulting suspension of routine outpatient follow-up. The non-randomized nature of the study introduces a high risk of residual confounding. Group allocation was time-based, which may induce information bias and could inflate the apparent effect of BVS monitoring, but formal protocols for ALS care and ventilatory management did not undergo major changes. Furthermore, all patients were managed within the same multidisciplinary program and under consistent criteria for MV initiation and follow-up, which mitigates the influence of temporal bias. Standard pharmacological management for ALS, including disease-modifying and symptomatic treatments, followed institutional protocols and did not differ between the BVS and non-BVS groups during the study period. Missing BVS data at specific visits were handled using an available-case approach without imputation. The lack of association between bulbar symptoms and survival should be interpreted with caution, given the potential selection bias related to the absence of domain-specific ALSFRS data. Although attrition was almost entirely due to death or tracheostomy, it remains possible that missing visits occurred more frequently in individuals with more advanced disease, and therefore some degree of informative censoring cannot be excluded. Taken together, the magnitude of the association observed should be interpreted with caution, even though the association between BVS monitoring and survival persisted after multivariable adjustment. Baseline ventilatory parameters were not uniform across subjects, as MV was initiated at different disease stages and individualized according to tolerance and clinical condition. This variability reflects real-world practice but precluded us from presenting a single set of initial parameters. Similarly, lung-function parameters [48] were not systematically collected after MV, due to limited feasibility and accuracy, particularly in bulbar subjects. Air stacking to measure maximal insufflation capacity [49] was also not evaluated, as it is not routinely performed in pressure support ventilation and is often unfeasible in bulbar involvement. Although more than one ventilator brand was used, no significant differences were observed between groups, and all devices belonged to the same generation of home MV platforms with comparable BVS capabilities. BVS-derived parameters were therefore interpreted mainly through within-patient longitudinal trends and waveform inspection.

Despite these limitations, this study provides novel and clinically relevant evidence. To our knowledge, it is the first to demonstrate an association between BVS-guided monitoring and improved survival in ALS subjects receiving MV. Furthermore, it highlights the prognostic significance of BVS-derived parameters. Collectively, these findings support the incorporation of BVS into routine ALS care and lay the foundation for future prospective multicenter studies with standardized ventilatory protocols and systematic longitudinal assessment of ventilatory and clinical parameters, which are warranted to confirm these findings and to more robustly address the limitations inherent to the present observational design.

## 5. Conclusions

In conclusion, BVS analysis represents a practical and effective tool for assessing patient–ventilator interaction in real-life conditions, without requiring additional testing for the patient. By enabling continuous, home-based monitoring, BVS provides clinicians with actionable data that reflect the patient’s respiratory status over time. In our cohort, the use of BVS was associated with improved survival and offered valuable prognostic information, allowing for more personalized, timely and forward-looking clinical decision-making. These findings should be interpreted in the context of potential period bias and technology drift related to the use of historical controls, and warrant confirmation in prospective contemporaneous studies.

## Figures and Tables

**Table 1:** General characteristics of the population and comparisons between the studied groups.

Variable		BVS Group (*n* = 57)	Non-BVS Group (*n* = 63)	*p*
Sex	Male (*n* = 85) Female (*n* = 35)	38 (66.7%) 17 (33.3%)	47 (74.6%) 16 (25.4%)	0.34
Site of onset	Bulbar (*n* = 35) Spinal (*n* = 85)	15 (26.3%) 42 (73.3%)	20 (31.7%) 43 (68.3%)	0.51
Age at diagnosis (years)	Mean (SD)	60.1 (11.3)	64.9 (11.1)	0.15
Mechanical ventilation	Noninvasive (*n* = 117) Invasive (*n* = 3)	54 (94.7%) 3 (100%)	63 (100%) 0 (0%)	0.06
Ventilator brand	Philips^®^ (*n* = 87)	45 (78.9%)	42 (66.7%)	0.13
	ResMed^®^ (*n* = 15)	8 (14%)	7 (11.1%)	0.63
	BREAS^®^ (*n* = 13)	4 (7%)	9 (14.3%)	0.20
	Other (*n* = 5)	0 (0%)	5 (7.9%)	
Interface model	Oronasal (*n* = 111)	51 (89.5%)	60 (95.2%)	0.23
	Nasal (*n* = 6)	3 (5.3%)	3 (4.8%)	0.90
	Tracheostomy (*n* = 3)	3 (5.3%)	0 (0%)	
Mechanical insuflation-exuflation device	Indication Infectiveness	48 (84.2%) 15 (26.3%)	47 (74.6%) 18 (28.6%)	0.20 0.78

BVS: built-in software, SD: standard deviation. Comparisons between groups were performed using the chi-square test or Fisher’s exact test for categorical variables, and Student’s *t*-test or Mann–Whitney U test for continuous variables, as appropriate. Percentages are calculated based on the total number of patients within each group (column percentages).

**Table 2:** Evolution of the studied variables during the follow-up of ALS patients in the BVS group treated with home MV.

Visits	1 (*n* = 57)	2 (*n* = 57)	3 (*n* = 56)	4 (*n* = 43)	5 (*n* = 32)	6 (*n* = 23)	7 (*n* = 10)	8 (*n* = 6)
ALSFRS-R scale (points), mean ± SD	26.5 ± 7.4	24.3 ± 8	21.5 ± 9.1	21.9 ± 8.5	20.0 ± 8.8	20.8 ± 8.8	20.2 ± 9.2	14.5 ± 6.8
Hours of MV use (hours) mean ± SD	7.50 ± 3.07	8.87 ± 4.21	11.02 ± 4.84	11.53 ± 5.15	11.87 ± 6.14	12.56 ± 6.87	13.60 ± 6.51	16.17 ± 7.10
Spontaneous inspirations (%), mean ± SD	70.17 ± 24.61	64.29 ± 26.08	61.63 ± 27.25	56.65 ± 29.89	53.84 ± 32.88	46.75 ± 34.60	50.82 ± 38.42	38.40 ± 37.29
Unintentional leak > 20 L/min (%)	33	23	39	26	34	22	33	33
Asynchronies (%)	53	51	43	42	44	26	42	33

**Table 3:** Multivariable Cox proportional hazards model evaluating the association between BVS monitoring and survival (*p* < 0.001).

Variable	Hazard Ratio	Std. Error	z	*p*-Value	95% CI
BVS group (vs. non-BVS)	0.18	0.04	−7.21	<0.001	0.11–0.27
Male (vs. female)	1.15	0.27	0.60	0.546	0.73–1.82
Mechanical insufflation–exsufflation (vs. no)	0.99	0.21	−0.05	0.957	0.65–1.51
Bulbar onset (vs. spinal)	1.07	0.25	0.32	0.687	0.69–1.68
≥64 years old at diagnosis (vs. <64 years)	0.83	0.19	−0.79	0.428	0.53–1.30

## Data Availability

The data supporting the findings of this study are available from the corresponding author upon reasonable request.

## Supplementary Materials

The following supporting information can be downloaded at https://www.mdpi.com/article/10.3390/jcm15041513/s1, Figure S1: National guidelines for initiation of non-invasive ventilation in neuromuscular disease; Figure S2: Patient flow and follow-up outcomes throughout the study; Figure S3: Percentage of patients with each type of asynchrony and its evolution during follow-up.

## Abbreviations

The following abbreviations are used in this manuscript:

ALS	Amyotrophic lateral sclerosis
BVS	Built-in ventilator software
MV	Mechanical ventilation
