# Supplementary material for: Impact of Built-In Software Monitoring on Survival in Amyotrophic Lateral Sclerosis Patients Receiving Home Mechanical Ventilation: A Cohort Study
Source: J Clin Med. 2026 Feb 14;15(4):1513. doi: 10.3390/jcm15041513 (PMC12941714; doi:10.3390/jcm15041513)
Supplement: Supplementary file 1 [file jcm-15-01513-s001.zip › jcm-4133949-supplementary.pdf]

Supplementary Materials

<u>**Follow up:**</u>

1. Slowly progressive neuromuscular diseases:

- Follow-up every 6–12 months.
- Assessment of symptoms, spirometry, arterial blood gases, SNIP/IPmax/EPmax, pulse oximetry.

2. Rapidly progressive neuromuscular diseases:

- Follow-up every 2–4 months.
- Assessment of symptoms, spirometry, peak cough flow, arterial blood gases, SNIP.
  In case of suspicion of nocturnal hypoventilation: nocturnal pulse oximetry (mean SpO_2, CT90, ODI), nocturnal transcutaneous capnography.
- Follow-up in a multidisciplinary clinic.

<u>**Criteria form mechanical ventilation**</u>

- Symptoms (e.g., orthopnea), nocturnal capnography with hypercapnia (Peak $PaCO_2 > 49$ mmHg), day capnography or arterial blood gas with hypercapnia ($PaCO_2 > 45$ mmHg in), nocturnal pulse-oximetry with desaturation (ODI >10 or CT90 >10%), forced vital capacity <50% of the predicted value, decline of pressure measures (SNIP, IPmax, EPmax).

Figure S1. Criteria for initiation of non-invasive mechanical ventilation in neuromuscular disease.

CT90: percentage of time with SpO_2 below 90%. IPmax: maximal inspiratory pressure. EPmax: maximal expiratory pressure. ODI: oxygen desaturation index. SNIP: sniff nasal inspiratory pressure.

Adapted from E. Farrero et al. Arch Bronconeumol. 2013;49(7):306–313 307.

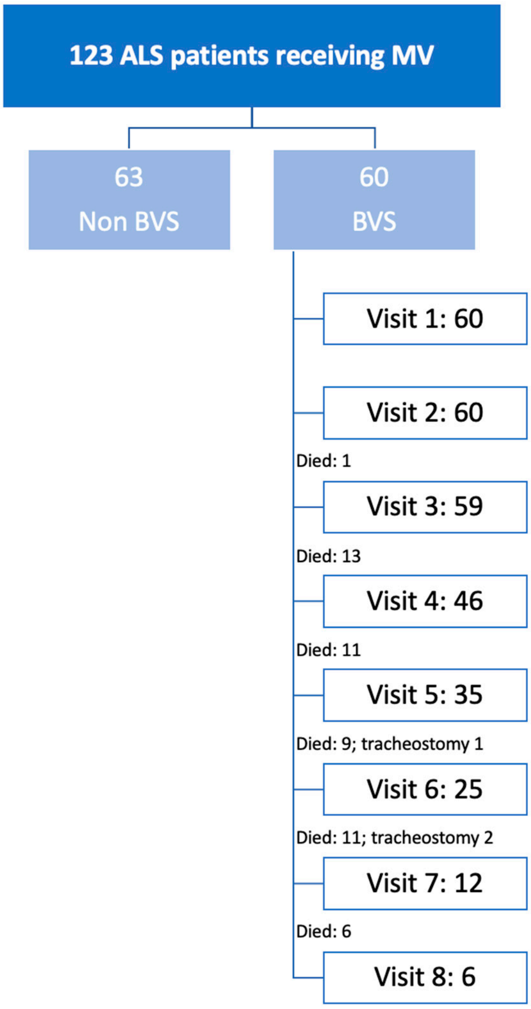

Figure S2. Patient Flow and Follow-Up Outcomes Throughout the Study

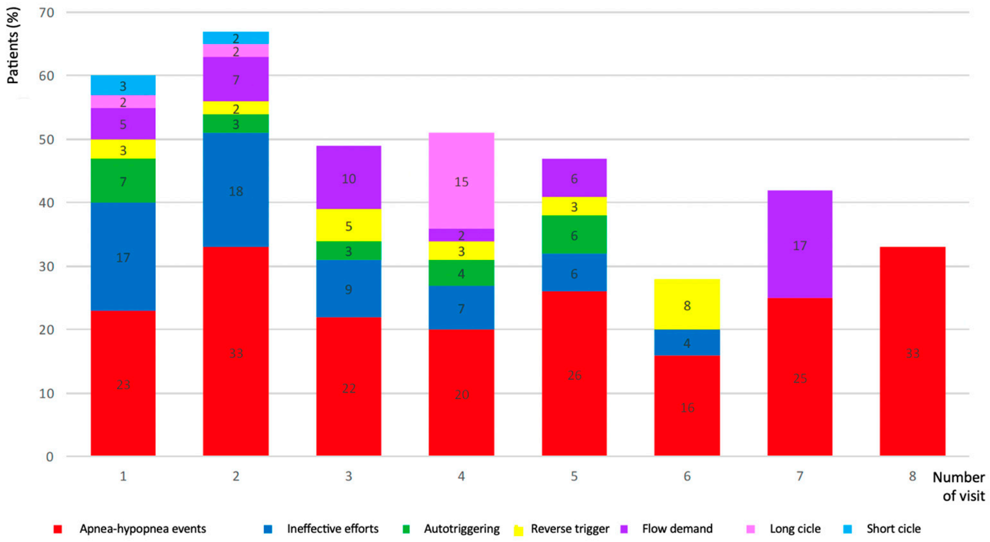

Figure S3. Percentage of patients with each type of asynchrony and its evolution during follow-up.
